# Developing a new sepsis screening tool based on lymphocyte count, international normalized ratio and procalcitonin (LIP score)

**DOI:** 10.1038/s41598-022-16744-9

**Published:** 2022-11-21

**Authors:** Bin Liu, Huimin Du, Jing Zhang, Jie Jiang, Xin Zhang, Faming He, Bailin Niu

**Affiliations:** 1 Department of Intensive Care Medicine, The First Affiliated Hospital of Chongqing Medical University, Youyi Road No.1, Yuzhong District, Chongqing, 400016 China; 2 Department of Intensive Care Medicine, Chongqing Emergency Medical Center, Chongqing University Central Hospital, School of Medicine, Chongqing University, Jiangkang Road No.1, YuZhong District, Chongqing, 400016 China; 3 Department of Oncology, The First Affiliated Hospital of Chongqing Medical University, Chongqing, 400016 China; 4 Greater Bay Area Institute of Precision Medicine (Guangzhou), Fudan University, Shanghai, 200433 China

**Keywords:** Infectious diseases, Diagnosis

## Abstract

Exploring an effective sepsis screening tool that can be widely implemented is important for improving the prognosis of sepsis worldwide. This study aimed to develop a new simple screening tool for sepsis (LIP scoring system) that includes the peripheral blood lymphocyte count, international normalized ratio, and procalcitonin level. In a single-center, prospective, observational study, 444 acute sepsis inpatients and 444 nonsepsis inpatients were ultimately included based on the Sepsis-3 and exclusion criteria. The differences in the Lym, INR, PCT level and other clinical biomarkers were compared between the two groups. Univariable and multivariable logistic regression analyses and receiver operating characteristic analysis were used to establish a LIP screening tool for sepsis with a combination of biomarkers. The Kappa and McNemar tests were used to evaluate the differences between the LIP screening results (LIP score ≥ 3) and Sepsis-3 criteria (SOFA score ≥ 2). Logistic regression analysis showed that the lymphocyte count, INR, PCT level, platelets, neutrophil/lymphocyte ratio (NLR) and prothrombin time (PT) were independent risk factors for the development of sepsis. The ROC analysis showed that the lymphocyte count, INR, and PCT level had high area under the ROC curve values (AUROC (95% CI): Lym 0.84 (0.810–0.860), INR 0.921 (0.902–0.938), PCT level 0.928 (0.909–0.944)). The LIP tool had satisfactory screening efficacy for sepsis (sensitivity, 92.8%; specificity, 94.1%), and a LIP score equal to or greater than 3 points had good agreement with Sepsis-3 criteria in the diagnosis of sepsis (Kappa = 0862 in the Kappa test and *P* = 0.512 in the McNemar test). The LIP tool has satisfactory sensitivity and specificity for sepsis screening, and it can be used for rapid screening of patients with sepsis in outpatient and emergency departments or in economically underdeveloped areas with limited resources.

## Introduction

Sepsis is defined as life-threatening organ dysfunction caused by a dysregulated host response to infection^1^. It is estimated that there are 31.5 million cases of sepsis and 19 million cases of severe sepsis worldwide each year, and approximately 5 million patients die from sepsis each year^2^. An early diagnosis of sepsis is closely related to an improved prognosis. On the basis of the existing Sepsis-3 criteria, the quick Sequential Organ Failure Assessment (qSOFA) was proposed as a screening tool for sepsis. The qSOFA has the advantages of being noninvasive, rapid, and easy to master; however, its sensitivity is not satisfactory^3^. For community health centers, emergency and outpatient clinics, and low- and middle-income countries and regions, the SOFA components proposed by the Sepsis-3 criteria are difficult to assess in a timely manner. Therefore, there is an urgent need to explore a new screening tool that can be used widely and rapidly reflect the status of the systemic inflammatory response in sepsis.

Sepsis is a syndrome of systemic inflammatory response due to infection^4^. Along with systemic inflammatory responses, compensatory anti-inflammatory responses are present and usually lead to suppression of host immune function^5^. A major feature of immunosuppression in sepsis is immune cell apoptosis, similar to the decreased levels of circulating lymphocytes^6^. Apoptosis, autophagy and other factors contribute to the early reduction in lymphocyte count^6–8^. In addition, procoagulant dysfunction is another important feature of sepsis, and many studies have revealed a close relationship between the infectious inflammatory response and coagulation function^9–12^. The activation of coagulation, activation of platelets, vascular endothelial cell damage, and release of inflammatory mediators all lead to the development of coagulopathy. Procoagulant and anticoagulant reactions are associated with the entire process of the systemic inflammatory response caused by infection. Clinically, a severe inflammatory response is often accompanied by abnormal coagulation function indicators and even disseminated intravascular coagulation^10,12–14^. Procalcitonin (PCT) is an endogenous peptide that is rapidly secreted in response to many inflammatory stimuli, is elevated in the early stages of infection (2–4 h) and peaks 12–24 h after infection, making it a timely and early biomarker of the pathophysiological state of sepsis infection^15,16^. Based on the pathophysiological characteristics of sepsis and many clinical case observations, we created the LIP (lymphocyte count, international normalized ratio, PCT level) tool as a simple early screening tool for sepsis.

The primary objective of this study was to investigate the screening efficacy of the LIP tool (LIP score ≥ 3 points) for sepsis and to compare it with the current Sepsis-3 criteria (SOFA score ≥ 2 points). The secondary objective was to perform a subgroup analysis of sepsis and investigate the diagnostic efficacy of the LIP tool in sepsis of both pulmonary and nonpulmonary origins.

## Materials and methods

### Study population

This was a prospective, observational study conducted at the First Affiliated Hospital of Chongqing Medical University. Patients with clinically suspected infection or who were hospitalized for infection between January 2018 and April 2021 were included in the study. The study was approved by the Ethics Committee of the First Affiliated Hospital of Chongqing Medical University and carried out in accordance with the Declaration of Helsinki. Written informed consent was obtained from all enrolled patients.

Adult patients (≥ 18 years) with acute infection or an acute exacerbation of a chronic infection were enrolled. The data were collected within 1 h of admission or within 1 h of an acute exacerbation for current inpatients, and patients were divided into sepsis and nonsepsis groups according to the Sepsis-3 definition. Patients with the following conditions were excluded from this study: (1) patients who had an unclear baseline SOFA score; (2) patients with a severe underlying disease (patients with active immunosuppression, such as those recently treated with radiotherapy, high-dose glucocorticoid or immunosuppressive therapy, severe chronic organ dysfunction, and cachexia); (3) patients with hematologic disorders affecting the lymphocyte count and patients taking oral anticoagulants that affect the INR (patients with lymphocytic leukemia or aplastic anemia and patients taking oral warfarin); and (4) patients who had a diagnosis of sepsis for > 24 h.

### Definition

#### Suspected infection

Suspected infection was defined as meeting any two of the following five conditions, which partly refers to a previous study^17^.

(A) General symptoms (fever, increased respiratory rate, decreased blood pressure or altered mental status, when cardiogenic or neurogenic factors were excluded).
(B) Specific symptoms of infection (respiratory system: cough, coughing purulent sputum; digestive system: abdominal pain, diarrhea, jaundice; urinary system: urinary frequency, urinary urgency, urinary pain; skin and soft tissue infection: local redness, swelling and heat).
(C) White blood cell (WBC) count greater than 12*10^9/L or less than 4*10^9/L.
(D) Confirmation by imaging findings.
(E) Antibiotic treatment and completion of body fluid culture immediately after admission.

#### High-dose glucocorticoid therapy

High-dose glucocorticoid therapy was defined as taking prednisone at a dosage > 1 mg/(kg*d) within 7 days prior to screening or switching to an equivalent or near-equivalent dosage.

#### Acute exacerbation

Acute exacerbation was specifically characterized by sudden onset of chills, high fever, increased heart rate and respiration, decreased blood pressure, and altered mental status due to infection in hospitalized patients.

#### LIP score

Based on univariable and multivariable logistic regression results and the efficacy of the combined Lym, INR and PCT diagnosis, a lymphocyte count (Lym) ≤ 1*10^9/L was defined as lymphocytopenia: a Lym between 0.7 and 1*10^9/L was defined as mild abnormality, and a Lym < 0.7*10^9/L was defined as severe abnormality, and these conditions were scored 1 and 2, respectively; an INR between 1.2 and 1.4 was considered mildly prolonged, and an INR > 1.4 was considered severely prolonged, and these conditions were scored 1 and 2 to show the distinction; a PCT level between 0.5 and 2 ng/ml was regarded as mildly elevated, a PCT level > 2 ng/ml was regarded as significantly elevated, and scores of 1 and 2 were assigned to show the difference. The LIP scoring system was established with the abovementioned corresponding scores. Based on clinical observations and an analysis of the preliminary data, we defined LIP scores ≥ 3 as a positive screening criterion for sepsis. Details of the LIP scores are shown in Table 1.

**Table 1:** LIP scoring system.

Items	Score		
	0	1	2
Lymphocyte count (*10^9/L)	> 1	0.7 ~ 1	< 0.7
International normalized ratio (INR)	< 1.2	1.2 ~ 1.4	> 1.4
Procalcitonin (PCT, ng/ml)	< 0.5	0.5 ~ 2	> 2

### Data collection

Arterial and venous blood samples were collected from all of the patients within the first hour of suspected infection in admitted patients or within 1 h of acute exacerbation in hospitalized patients, and the components of the SOFA score and data required for this study were obtained simultaneously. Basic patient information was collected. The clinical information that was collected is shown in Table 2.

**Table 2:** Population and clinical characteristics.

Items	All patients (N = 888)	Sepsis group (N = 444)	Nonsepsis group (N = 444)	*P* value
**Characteristics**				
Age, median (IQR)	60(44–72)	67 (55–76)	49(32–65)	< 0.001*
Sex, male, n (%)	497(56.0)	275 (61.9)	222 (50.0)	< 0.001*
**Clinical scoring**				
qSOFA ≥ 2, n (%)	207(23.3)	196 (44.1)	11 (2.5)	< 0.001*
SIRS ≥ 2, n (%)	613(69.0)	374 (84.2)	239 (53.8)	< 0.001*
APACHE II score, median (IQR)	13(7–18)	17 (14–21)	8 (6–12)	< 0.001*
SAPS II, median (IQR)	22(13–33)	33(24–43)	13(7–20)	< 0.001*
**Outcome**				
Mortality, n (%)	125(14.1%)	123 (27.7)	2 (0.5)	< 0.001*
**Biomarker**				
Lym, median (IQR)	0.74(0.48–1.21)	0.54 (0.37–0.73)	1.11 (0.77–1.57)	< 0.001*
PLT, median (IQR)	185(126–258)	137 (85–211)	218 (175–290)	< 0.001*
WBC, median (IQR)	10.21(6.53–14.47)	11.00 (6.87–16.29)	9.73 (6.45–12.68)	< 0.001*
N#, median (IQR)	8.40(4.98–12.77)	9.93 (6.02–15.05)	7.29 (4.42–10.22)	< 0.001*
NP, median (IQR)	0.86(0.77–0.91)	0.91 (0.86–0.93)	0.80 (0.70–0.86)	< 0.001*
NLR, median (IQR)	11.49(5.57–22.35)	19.55 (11.13–29.25)	6.41 (3.59–11.70)	< 0.001*
PT, median (IQR)	14.20(12.90–16.20)	16.20 (14.83–17.70)	13.10 (12.20–13.90)	< 0.001*
APTT, median (IQR)	37.45(31.40–44.85)	41.90 (35.43–49.85)	33.75 (28.73–38.58)	< 0.001*
INR, median (IQR)	1.14(1.04–1.32)	1.32 (1.23–1.49)	1.05 (1.00–1.11)	< 0.001*
PCT, median (IQR)	0.54(0.09–9.73)	9.19 (1.58–51.42)	0.11 (0.05–0.31)	< 0.001*
Lac, median (IQR)	1.5(1.0–2.5)	2.27 (1.40–4.40)	1.20 (0.80–1.60)	< 0.001*
**Site of infection**				
Pneumonia, n (%)	287(32.3)	147 (33.1)	140 (31.5)	N/A
Urinary tract infection, n (%)	116(13.0)	68 (15.3)	48 (10.8)	N/A
Gastrointestinal perforation, n (%)	59(6.6)	48 (10.8)	11 (2.5)	N/A
Skin and soft tissue infections, n (%)	76(8.6)	37 (8.3)	39 (8.8)	N/A
Biliary tract infection, n (%)	38(4.3)	37 (8.3)	1 (0.2)	N/A
Liver abscess, n (%)	30(3.4)	23 (5.2)	7 (1.6)	N/A
Intestinal infection, n (%)	84(9.5)	30(6.8)	54(12.2)	N/A
Mediastinal infection, n (%)	7(0.8)	7 (1.6)	0	N/A
Appendicitis, n (%)	65(7.3)	4 (0.9)	61 (13.7)	N/A
Cholecystitis, n (%)	14(1.6)	3 (0.7)	11 (2.5)	N/A
URTI, n (%)	38(4.3)	0	38 (8.6)	N/A
Other, n (%)	74(8.3)	40 (9)	34 (7.6)	N/A
**Admission department**				
ICU, n (%)	408(45.9)	358 (80.6)	50 (11.3)	< 0.001*
Ward (%)	480(54.1)	86 (19.4)	394 (88.7)	
**Bacteriological culture**				
Blood, n (%)	134(15.1)	130(29.3)	4(0.9)	< 0.001*
Urine, n (%)	42(4.7)	32(7.2)	10(2.2)	
Sputum and other, n (%)	71(8.0)	50(11.2)	21(4.7)	

*qSOFA* Quick Sequential Organ Failure Assessment; *SIRS* Systemic inflammatory response syndrome; *APACHE II* Acute Physiology and Chronic Health Evaluation II; *SAPS II* Simplified Acute Physiology Score II; *Lym* lymphocyte count; *N#* Absolute neutrophil count; *NP* Neutrophil percentage; *WBC* White blood cell; *NLR* Neutrophil-to-lymphocyte ratio; *PLT* Platelet; *PCT* Procalcitonin; *PT* Prothrombin time; *APTT* Activated partial thrombin time; *INR* International normalized ratio; *Lac* Lactic acid; *URTI* Upper respiratory tract infection. * indicates a significant value, *P* < 0.05.

### Statistical analysis

SPSS 25.0 (IBM Corp.) and MedCalc statistical software 20.0.3.0 (MedCalc Software, Belgium) were used for statistical analysis. Data were tested for normality using the one-sample Kolmogorov–Smirnov test, and normally and nonnormally distributed continuous variables were described as the mean (standard deviation) and median (interquartile spacing), respectively. For continuous variables, a t test was used to analyze normally distributed data, and the Mann–Whitney U test was used for nonnormally distributed data. The chi-square test was used for categorical data. The effect of each biomarker on the presence of sepsis was analyzed by univariable and multivariable logistic regression, and the screening performance of each biomarker for sepsis was compared by the area under the ROC curve. The McNemar test was performed to assess the difference between the LIP tool and Sepsis-3 criteria in the diagnosis of sepsis. The Kappa test was performed to assess the agreement between the LIP tool and Sepsis-3 criteria in the diagnosis of sepsis. A *P* value < 0.05 was considered to indicate statistically significant differences.

### Ethics approval and consent to participate

This study was approved by our local ethics review committee in compliance with the Declaration of Helsinki. Written informed consent was obtained from all of the patients enrolled in the study (Ethics Committee of the First Affiliated Hospital of Chongqing Medical University, Chongqing, China, ethical document NO. 2019–312).

## Results

### Study population and their clinical characteristics

**Figure 1:**
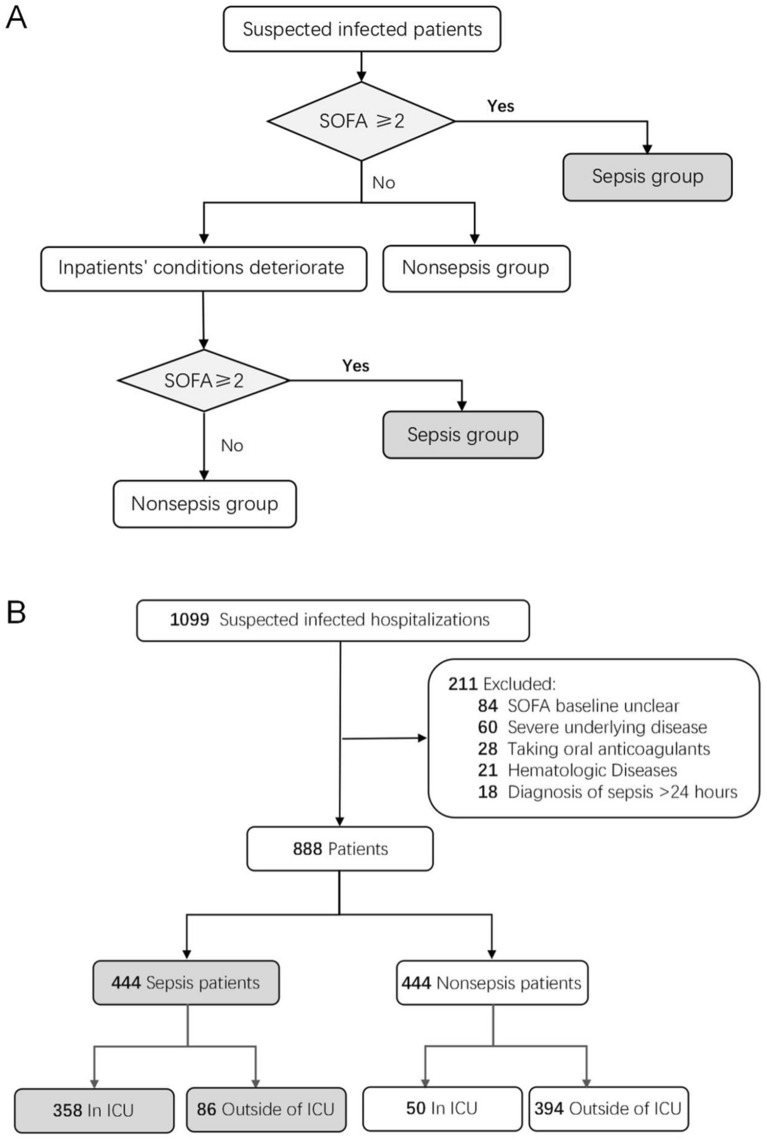
Flowchart of study participant enrollment. (**A**) The judgment process for sepsis in patients with suspected infection. SOFA ≥ 2 means that the SOFA score increases by 2 or more points compared to the base value. (**B**) Sepsis and nonsepsis patient cohort enrollment process.

A total of 1099 hospitalized patients with suspected infection were enrolled. After application of the exclusion criteria, 444 sepsis and 444 nonsepsis patients were included in the study based on the Sepsis-3 criteria. The enrollment process of the investigated subjects is shown in Fig. 1. The corresponding clinical characteristics of each study group are shown in Table 2.

### Logistic regression analysis of the biomarkers in the diagnosis of sepsis

The univariable logistic regression analysis showed that Lym, INR, PCT, NLR, PLT, PT, N#, NP, WBC, APTT and Lac levels were significant factors in the diagnosis of sepsis (*p* < 0.05, Table 3). Furthermore, the multivariable logistic regression analysis showed that Lym (OR 0.178, 95% CI 0.068–0.465), INR (OR 1.120, 95% CI 1.067–1.176), PCT (OR 1.101, 95% CI 1.041–1.165), NLR (OR 1.078, 95% CI 1.042–1.114), PLT (OR 0.995, 95% CI 0.992–0.998) and PT (OR 1.473, 95% CI 1.008–2.153) were independent risk factors for sepsis (*p* < 0.05, Table 3).

**Table 3:** Univariable and multivariable logistic regression analysis of included biomarkers.

	Univariable analysis		Multivariable analysis	
	OR (95% CI)	*P* value	OR (95% CI)	*P* value
Lym	0.040(0.025–0.064)	< 0.001*	0.178(0.068–0.465)	< 0.001*
INR	1.179(1.153–1.205)	< 0.001*	1.120(1.067–1.176)	< 0.001*
PCT	1.492(1.363–1.634)	< 0.001*	1.101(1.041–1.165)	0.001*
NLR	1.137(1.115–1.160)	< 0.001*	1.078(1.042–1.114)	< 0.001*
PLT	0.992(0.991–0.994)	< 0.001*	0.995(0.992–0.998)	0.001*
PT	3.316(2.828–3.888)	< 0.001*	1.473(1.008–2.153)	0.046*
N#	1.109(1.081–1.139)	< 0.001*		
NP	1.139(1.116–1.163)	< 0.001*		
WBC	1.068(1.044–1.093)	< 0.001*		
APTT	1.097(1.078–1.116)	< 0.001*		
Lac	2.986(2.451–3.637)	< 0.001*		
Sex	0.615(0.471–0.803)	< 0.001*		
Age	1.051(1.042–1.060)	< 0.001*		

*OR* Odds ratio; *CI* Confidence interval; *Lym* Lymphocyte count; *N#* Absolute neutrophil count; *NP* Neutrophil percentage; *WBC* White blood cell count; *NLR* Neutrophil-to-lymphocyte ratio; *PLT* Platelet count; *PCT* Procalcitonin; *PT* Prothrombin time; *APTT* Activated partial thrombin time; *INR* International normalized ratio; *Lac* Lactic acid. * indicates a significant value, *P* < 0.05.

### Performance of individual biomarkers in the screening of sepsis

**Figure 2:**
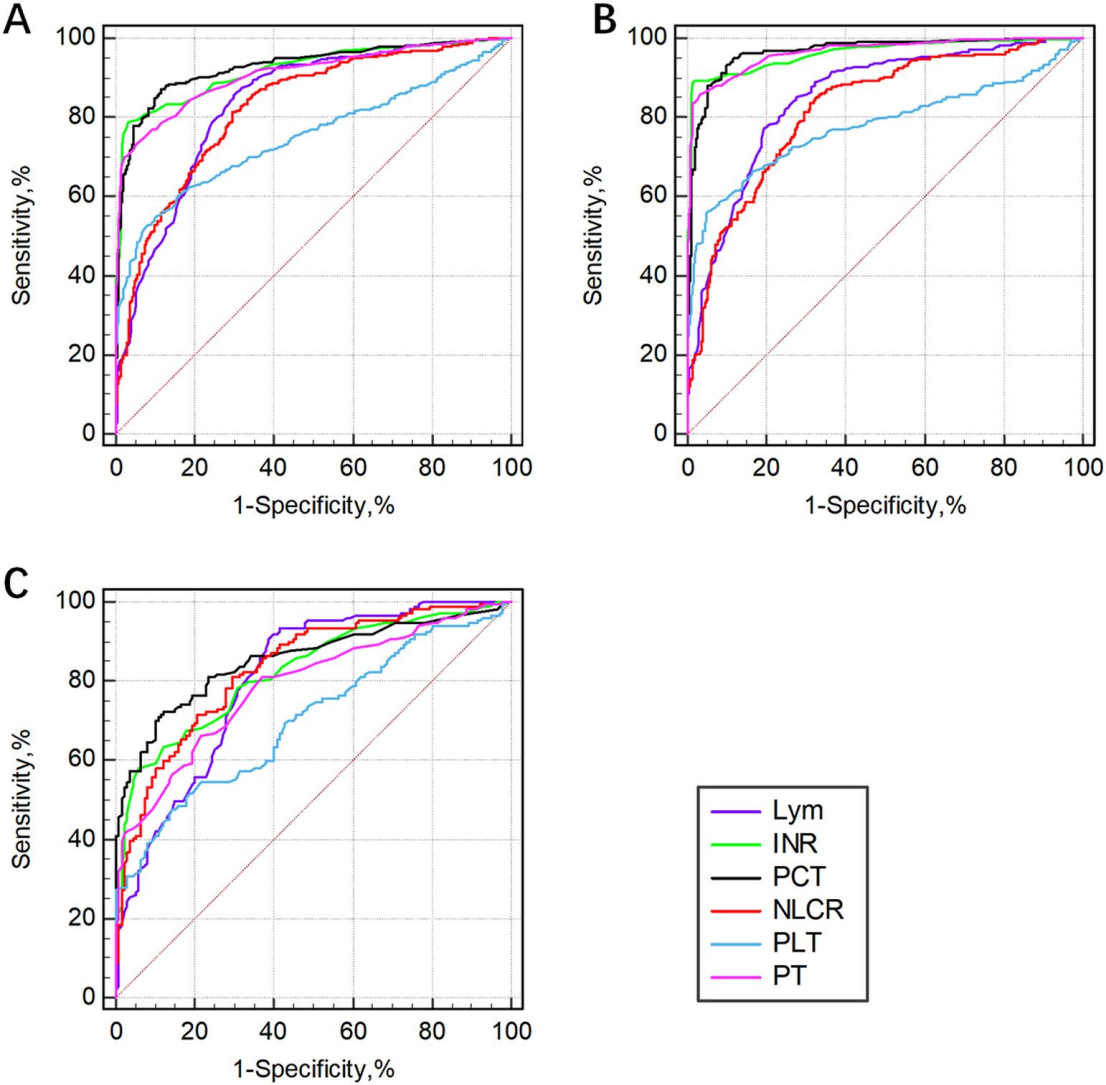
The performance of individual biomarkers in the screening of sepsis was evaluated by ROC analysis. (**A**): All patients; (**B**): Nonpulmonary infection group; (**C**): Pulmonary infection group. *Lym* lymphocyte count; *INR* international normalized ratio; *PCT* Procalcitonin; *NLR* Neutrophil-to-lymphocyte ratio; *PLT* Platelet count; *PT* Prothrombin time.

The screening efficacy of the different biomarkers for sepsis as evaluated by the ROC curves are presented in Fig. 2 and Table 4. Among all patients, the PCT level had the highest screening efficacy, followed by the PT, INR and Lym, with an AUROC of more than 80%. Among them, the highest sensitivity was 86.7% when the optimal cutoff value of the PCT level was 0.62, and the highest specificity was 96.8% when the optimal cutoff value of the INR was 1.20. In the subgroup of patients with pulmonary infection, the PCT level also had the highest screening efficiency, followed by the NLR, INR and Lym, and the AUROC was more than 80%. The highest sensitivity was 90.5% when the optimal cutoff value of Lym was 0.84, and the highest specificity was 94.3% when the optimal cutoff value of the INR was 1.19. In the nonpulmonary infection subgroup, the PCT level still had the highest screening efficiency, followed by the INR, PT, LYM and NLR. The highest sensitivity was 94.9% when the optimal cutoff value of the PCT level was 0.59, and the highest specificity was 98.0% when the optimal cutoff value of the INR was 1.22.

**Table 4:** Performance of individual indicators in the screening of sepsis.

	Lym	INR	PCT	NLR	PLT	PT
**All patients**						
Cutoff value	0.84	1.20	0.62	10.04	147	14.9
AUROC (95% CI)	0.836 (0.810–0.860)	0.921 (0.903–0.940)	0.928 (0.909–0.944)	0.826 (0.799–0.850)	0.750 (0.720–0.778)	0.909 (0.888–0.927)
Sensitivity (%)	85.4	78.8	86.7	81.3	54.5	73.2
Specificity (%)	70.3	96.8	88.7	70.7	90.3	94.8
**Pulmonary infection**						
Cutoff value	0.84	1.19	0.70	9.98	148	14.1
AUROC (95% CI)	0.805 (0.754–0.849)	0.824 (0.775–0.867)	0.854 (0.808–0.893)	0.832 (0.784–0.874)	0.701 (0.644–0.753)	0.788 (0.736–0.834)
Sensitivity (%)	90.5	57.8	70.1	81.0	47.6	66.0
Specificity (%)	61.4	94.3	90.0	70.7	85.7	78.6
**Nonpulmonary infection**						
Cutoff value	0.75	1.22	0.59	8.74	140	14.9
AUROC (95% CI)	0.850 (0.819–0.878)	0.962 (0.943–0.976)	0.966 (0.949–0.979)	0.823 (0.790–0.853)	0.779 (0.744–0.812)	0.965 (0.947–0.978)
Sensitivity (%)	77.1	89.2	94.9	85.9	56.2	85.9
Specificity (%)	80.6	98.0	88.2	66.8	94.7	96.7

*Lym* Lymphocyte count; *INR* International normalized ratio; *PCT* Procalcitonin; *NLR* Neutrophil-to-lymphocyte ratio; *PLT* Platelet count; *PT* Prothrombin time; *AUROC (95% CI)* Area under the receiver operating characteristic curve (95% confidence interval). * indicates a significant value, *P* < 0.05.

### Efficacy of combined biomarkers in screening for sepsis

**Figure 3:**
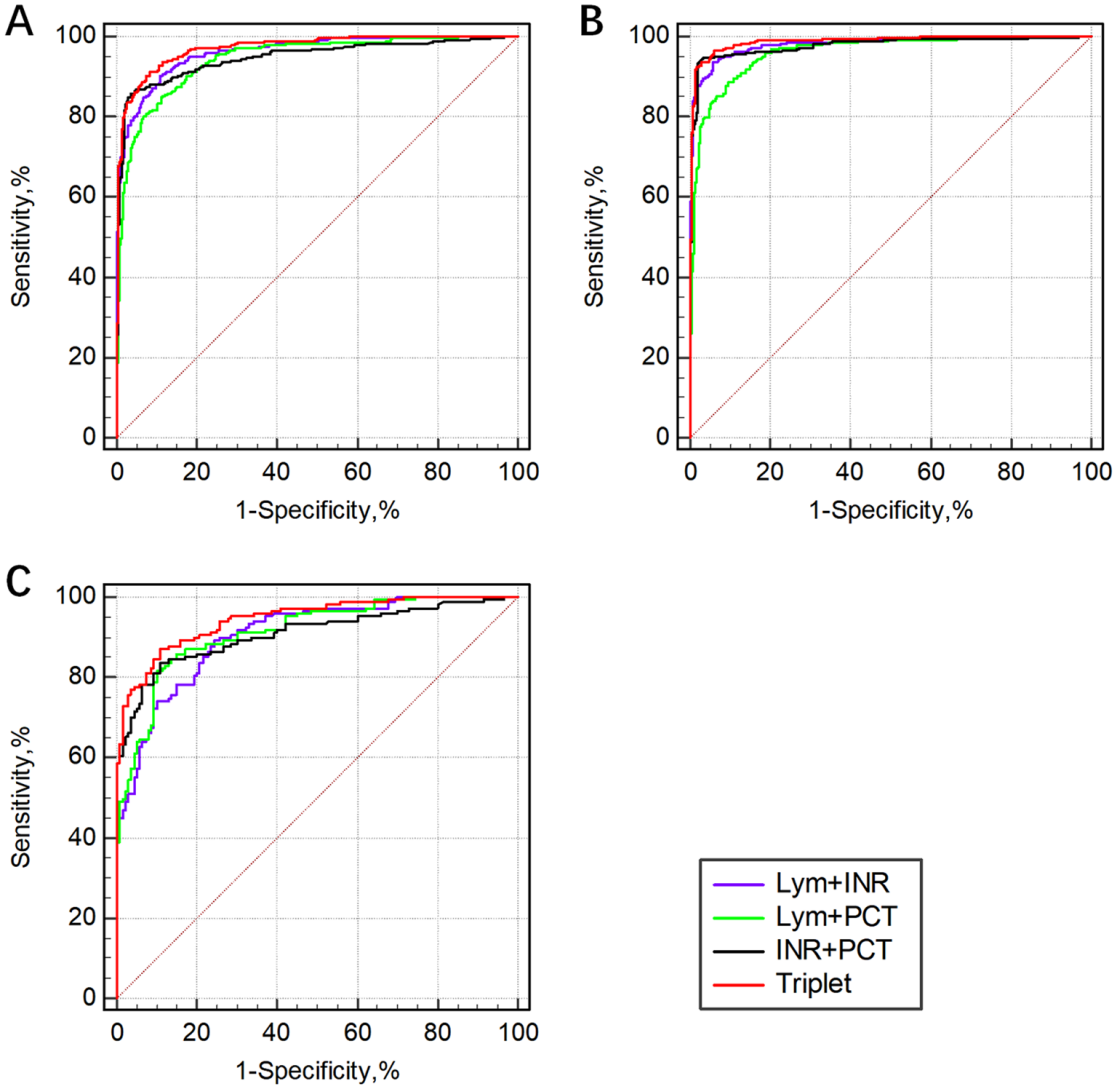
The combined diagnostic efficacy of the biomarkers for sepsis as evaluated by ROC curves. (**A**): All patients; (**B**): Nonpulmonary infection group; (**C**): Pulmonary infection group. *Lym* Lymphocyte count; *INR* International normalized ratio; *PCT* Procalcitonin.

Based on univariable and multivariable logistic regression analysis and the results of screening efficacy of individual biomarkers for sepsis, the screening efficacies of the combination of biomarkers (Lym, INR and PCT level) included in the LIP tool for sepsis, as evaluated by the ROC curves, are presented in Fig. 3 and Table 5. The screening efficacy of the combination of the three biomarkers (Lym plus INR plus PCT level) was higher than that of any two biomarkers, with an AUROC of 0.973 (95% CI: 0.960–0.982), 0.946 (95% CI: 0.913–0.969) and 0.988 (95% CI: 0.976–0.995) in all patients, pulmonary infection patients and nonpulmonary infection patients, respectively. In patients with nonpulmonary infection, the screening efficacy of any two of the three biomarkers combined was higher than that in patients with pulmonary infection.

**Table 5:** Efficacy of combined biomarkers in the screening of sepsis.

	Lym + INR	Lym + PCT	INR + PCT	Triplet
**All patients**				
AUROC (95% CI)	0.962 (0.947–0.974)	0.944 (0.927–0.958)	0.948 (0.931–0.961)	0.973 (0.960–0.982)
**Pulmonary infection**				
AUROC (95% CI)	0.906 (0.866–0.937)	0.914 (0.875–0.943)	0.909 (0.869–0.940)	0.946 (0.913–0.969)
**Nonpulmonary infection**				
AUROC (95% CI)	0.981 (0.967–0.990)	0.960 (0.941–0.974)	0.977 (0.962–0.988)	0.988 (0.976–0.995)

*Lym* Lymphocyte count; *INR* International normalized ratio; *PCT* Procalcitonin; *AUROC (95% CI)* area under the receiver operating characteristic curve (95% confidence interval). Triplet = Lym + INR + PCT.

### Performance of the LIP score in sepsis screening compared with the APACHE II score and SAPS II

**Figure 4:**
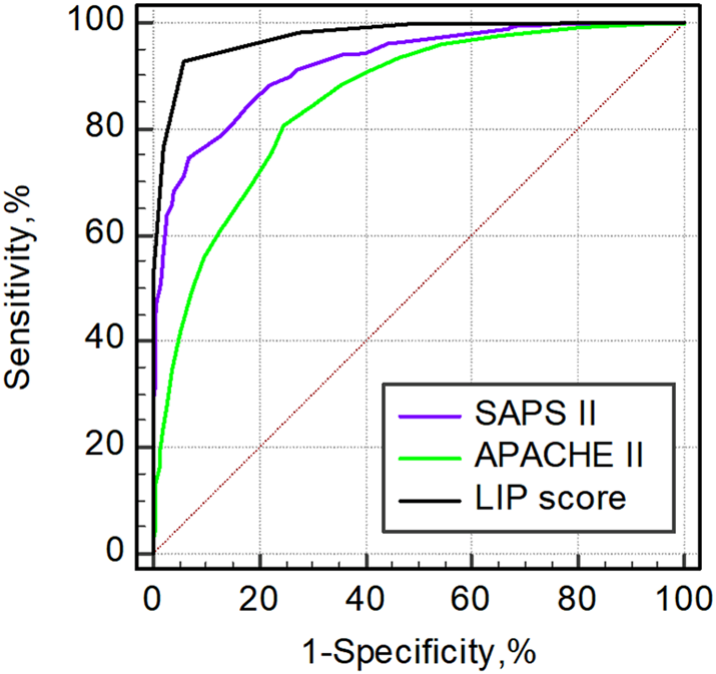
Performance of the LIP score in sepsis screening compared with the APACHE II score and SAPS II. The AUROCs of the SAPS II, APACHE II score, and LIP score were 0.922 (95% CI, 0.902–0.939), 0.853 (95% CI, 0.828–0.876), and 0.974 (95% CI, 0.961–0.983), respectively. *AUROC (95% CI)* Area under the receiver operating characteristic curve (95% confidence interval); *APACHE II* Acute Physiology and Chronic Health Evaluation II; *SAPS II* Simplified Acute Physiology Score II.

Figure 4 shows the power of the LIP score in sepsis screening and a comparison with the Acute Physiology and Chronic Health Evaluation II (APACHE II) score and the Simplified Acute Physiology Score II (SAPS II). The LIP score had the highest AUROC of 0.974 (95% CI: 0.961–0.983), while the APACHE II score and SAPS II had AUROC values of 0.853 (95% CI: 0.828–0.876) and 0.922 (95% CI: 0.902–0.939), respectively. Moreover, the optimal cutoff value of the LIP score in sepsis screening was 3, because the AUROC of the LIP score was highest when the score was greater than or equal to 3 (sFig. 1), and the sum of the sensitivity, specificity, positive predictive value, and negative predictive value were also the highest (sFig. 2).

### Screening power of a LIP score ≥ 3 for sepsis and its agreement with a SOFA score ≥ 2

For all patients, when the LIP score was greater than or equal to 3, the sensitivity, specificity, positive predictive value, negative predictive value and diagnostic odds ratio of sepsis screening were 92.8%, 94.1%, 94.1%, 92.9% and 196.6, respectively. In the subgroup analysis, LIP scores greater than or equal to 3 were more effective in the screening of nonpulmonary sepsis than in that of pulmonary sepsis. Nevertheless, their diagnostic odds ratios were all over 50. In addition, there was good agreement between the effectiveness of the LIP score ≥ 3 and SOFA score ≥ 2 in screening for sepsis (Kappa = 0862 in the Kappa test, and *P* = 0.512 in the McNemar test) (Table 6).

**Table 6:** Screening power of a LIP score ≥ 3 for sepsis and its consistency with a SOFA score ≥ 2.

	Sensitivity (%)	Specificity (%)	PPV (%)	NPV (%)	+LR	−LR	OR	Kappa	*P* value
All patients	92.8	94.1	94.1	92.9	15.73	0.08	196.6	0.869	0.512
Pulmonary infection	84.4	90.7	90.5	84.7	9.08	0.17	53.4	0.749	0.132
Nonpulmonary infection	97.0	95.7	95.7	97.0	22.56	0.03	752.0	0.927	0.523

*PPV* Positive predictive value; *NPV* Negative predictive value; + *LR* Positive likelihood ratio; − *LR* Negative likelihood ratio; *OR* odds ratio. Kappa values were obtained by the Kappa test, and the *P* value was obtained by the McNemar test.

## Discussion

Early diagnosis and treatment can improve the prognosis of sepsis^18^. We screened common biomarkers used for early diagnosis of sepsis and found that a new combination of the Lym, INR, and PCT was highly accurate for sepsis diagnosis in patients with acute infection. The LIP tool was developed based on the pathophysiological nature of sepsis-induced immunosuppression, procoagulant dysfunction, and the systemic inflammatory response, and the three biomarkers included in the LIP scoring system can be routinely assessed at most medical institutions worldwide. Many medical institutions can also carry out point-of-care testing (POCT), which greatly shortens the screening time for sepsis^19–22^. The combination of multiple biomarkers has complementary advantages, and ROC analysis showed that the LIP scoring tool has satisfactory screening efficacy. To eliminate the bias caused by nonacute infectious factors and some other unclear factors as much as possible, stricter exclusion criteria were set in this study. Admittedly, such exclusion criteria also narrow the range of patients who can screen be screened with the LIP tool to some extent.

Due to the complex mechanisms of sepsis and the complex patient baseline background, it is also difficult to develop an ideal sepsis screening tool that can be used without any additional control conditions. In this study, patients with unclear SOFA scores at baseline and those with severe underlying diseases, immunosuppression, severe chronic organ dysfunction, or cachexia were excluded. First, these disease states tend to bias the detection value of biomarkers. In addition, patients with severe underlying diseases are often not the group that relies most on screening tools, and severe underlying diseases themselves usually require hospitalization for the completion of a comprehensive evaluation. Patients with sepsis diagnosed more than 24 h prior to enrollment were excluded because they had already been diagnosed with sepsis and received relevant treatment, which can change the levels of the investigated biomarkers; this could easily cause bias in the results of this study. In addition, because such patients were diagnosed with sepsis, they no longer required screening.

Subgroup analysis of pulmonary and nonpulmonary infections was performed in our study. Patients with pulmonary infection usually receive adequate attention from a clinician in the early stage of disease because their clinical manifestations (fever, shortness of breath, etc.) are often more pronounced, and their treatment is often timely; the shock and mortality rates of such patients are generally lower than those of patients with nonpulmonary infections, such as abdominal and urinary tract infections^23,24^. A retrospective study in Hungary found that the rates of pulmonary infections leading to severe sepsis and septic shock were 12.9% and 14.8%, the rates of abdominal infections leading to severe sepsis and septic shock were 22.9% and 37.7%, and the rates of urinary tract infections leading to severe sepsis and septic shock were 25.7% and 14.8%, respectively^23^. The LIP scoring system has a relatively high screening efficiency for pulmonary sepsis, and it has a higher screening efficiency for nonpulmonary sepsis, which is more helpful for actual clinical practice.

Sepsis is the leading cause of lymphocytopenia among hospitalized patients^6^, and lymphocytopenia is associated with increased mortality^25,26^. A retrospective study conducted in Spain showed that patients with community-acquired pneumonia and sepsis combined with lymphocytopenia had higher rates of ICU admission and mortality^27^. A UK cohort study of 40909 individuals showed that lymphopenia was an independent predictor of mortality from primary care pneumonia and that even low-normal lymphocyte counts were associated with increased mortality in the short or long term compared with higher lymphocyte counts^28^. A meta-analysis showed that patients with lymphocytopenia had a threefold increased risk of developing severe sepsis^29^. In our study, the peripheral blood lymphocyte count was found to have good sensitivity (85.4%) for the diagnosis of sepsis, especially for sepsis of pulmonary origin (90.5%), with an optimal cutoff value of 0.84. However, the specificity of the peripheral blood lymphocyte count for the diagnosis of sepsis of pulmonary origin was relatively poor (61.4%); therefore, it needs to be combined with other biomarkers to improve its specificity. In recent years, the neutrophil-to-lymphocyte ratio (NLR) has also been studied for the diagnosis of sepsis^30,31^. There is too much variation in the optimal threshold of the NLR for diagnosing sepsis proposed by many recent studies because the NLR ranges from 4.36 to 23.28, making it difficult to provide a reference for practical clinical decision-making^32^. In this study, the area under the ROC curve was 0.826 when the NLR cutoff value was 10.04, at which point the sensitivity was 81.3% and the specificity was 70.7%.

During the development of sepsis, inflammation and activation of the coagulation system are important responses in host defense^12^. Endothelial cell dysfunction and abnormalities of the anticoagulation system are hallmarks of sepsis-related coagulation dysfunction^13^. On the basis of a previous study^33^ and clinical observations, we defined 1.2 ≤ INR ≤ 1.4 as a mild abnormality and an INR > 1.4 as a severe abnormality. Lyons et al. found an increase in sepsis mortality among patients with a prolonged INR secondary to sepsis-related coagulation abnormalities^33^. Liu et al. found that the INR was an independent risk factor for the 28-day mortality of sepsis^34^. Here, when the cutoff value was 1.19, the sensitivity, specificity and AUROC of INR were 78.8%, 96.8% and 0.921, respectively. The low sensitivity was due to the low proportion of patients with prolonged coagulation function in the group with pulmonary infection (sensitivity 57.8% and AUROC 0.824) at the time of initial sepsis diagnosis, whereas the proportion was very high in the group with nonpulmonary sepsis (sensitivity 89.2% and 0.980). Therefore, INR alone as a screening tool for sepsis has limitations.

In clinical practice, the PCT level is useful for the early diagnosis of sepsis^35,36^. The more current guidelines and consensus suggest that severe infection should be considered when the PCT level is > 0.5 ng/ml. Cabral et al. found that the best sensitivity and specificity for sepsis diagnosis was achieved when the PCT level threshold was 0.5 ng/ml^37^. It has also been reported that a moderate systemic inflammatory response syndrome should be considered when the PCT level is 0.5–2 ng/ml, and when the PCT level is 2–10 ng/ml, sepsis is most likely; moreover, sepsis or septic shock is almost always present when the PCT level is > 10 ng/ml^38^. A recent meta-analysis and systematic review also showed that for neonatal sepsis, a PCT cutoff value of 0.5–2 ng/ml is a more appropriate diagnostic interval^39^. In this study, the optimal cutoff value of PCT was 0.62 with an AUROC of 0.928 for all patients. Therefore, the use of the PCT level (0.5–2 ng/ml) alone as a screening tool is not very satisfactory. For the combination of the Lym, INR and PCT level in screening for sepsis, the AUROC reached 0.973 (96% CI: 0.960–0.982) in all patients. However, we also found that the screening value of a single indicator was limited for the pulmonary infection subgroup, which is a prominent feature of pulmonary infection-derived sepsis. When any two of the three biomarkers were combined, the AUROC all exceeded 90%, whether for pulmonary or nonpulmonary infections. The combined AUROC of the three indicators for sepsis screening was the largest in both the pulmonary infection group and nonpulmonary infection group (Table 5 and sTable 1).

Thrombocytopenia is common in sepsis patients, and the SOFA score uses platelet count as an indicator of coagulation function. The normal range of the platelet count is wide (100–300), which to some extent can lead to a lack of sensitivity. Here, the screening efficacy of the platelet count alone was not satisfactory, with an AUROC of 0.750 at an optimal cutoff value of 147 when the sensitivity was 54.5% and the specificity was 90.3%. This study also revealed that PT is an independent risk factor for sepsis. Because the essence of the INR and PT is the same, the INR is comparable in different laboratories. That is why the INR was selected for inclusion in the LIP tool rather than PT. In recent years, more novel sepsis biomarkers, such as HMGB-1, CD64, IL-6, heparin-binding protein (HBP) and triggering receptors expressed on myeloid cells-1 (TREM-1), have been identified. They are also expected to exhibit better screening performance in the future.

The present study has the following advantages. First, the three indicators included in the LIP tool can reflect the pathophysiological characteristics of the immunosuppressed, uncontrolled systemic inflammatory response state of sepsis, are complementary in clinical screening and diagnosis, and can reach a relatively satisfactory sensitivity and specificity. Second, the three indicators included in the LIP tool are commonly used indicators that are widely measured in clinical practice and can be used in the majority of acute outpatient clinics and in economically challenged areas. Assessment of the three indicators can be completed within one hour, which allows the administration of antibiotics within one hour. Third, in screening for sepsis, the LIP tool is simpler to use than the SOFA score, and this study showed good agreement between the LIP tool and the existing Sepsis-3 criteria for the diagnosis of sepsis.

There are also some limitations of this study. First, this was a single-center study, and the next step is to move forward with a multicenter study. Second, because of the exclusion criteria in this study, the scope of the LIP tool was reduced, and the next step will be to study the adjusted LIP score in patients with different serious underlying diseases, such as those taking anticoagulants or glucocorticoids.

## Conclusion

The proposed LIP tool (LIP scoring system) has satisfactory clinical screening efficacy based on the exclusion of interfering underlying diseases and is in good agreement with the SOFA score. As the three biomarkers included in the LIP score are commonly assessed in clinical practice, they are suitable for screening sepsis patients worldwide and can be used in a wide range of acute outpatient clinics and in economically disadvantaged areas. The LIP tool is a low-cost, simple, and efficient screening tool.

## Supplementary Information

Supplementary Information.

## Data Availability

The data that support the findings of this study are available from the corresponding author upon reasonable request.

## Abbreviations

APACHE II: Acute Physiology and Chronic Health Evaluation II
SAPS II: Simplified Acute Physiology Score II
APTT: Activated partial thrombin time
AUROC: Area under the receiver operating characteristic curve
CI: Confidence interval
INR: International normalized ratio
Lac: Arterial lactic acid
Lym: Lymphocyte count
N#: Neutrophil
NLR: Neutrophil-to-lymphocyte ratio
NP: Neutrophil percentage
NPV: Negative predictive value
OR: Odds ratio
PCT: Procalcitonin
PLT: Platelet count
PPV: Positive predictive value
PT: Prothrombin time
qSOFA: Quick Sequential Organ Failure Assessment
SOFA: Sequential Organ Failure Assessment
WBC: White blood cell count
+LR: Positive likelihood ratio
−LR: Negative likelihood ratio
