# Supplementary material for: Developing a new sepsis screening tool based on lymphocyte count, international normalized ratio and procalcitonin (LIP score)
Source: Sci Rep. 2022 Nov 21;12:20002. doi: 10.1038/s41598-022-16744-9 (PMC9678875; doi:10.1038/s41598-022-16744-9)
Supplement: Supplementary file 1 — Supplementary Information. [file 41598_2022_16744_MOESM1_ESM.docx]

**Supplementary Content**

**sFigure 1. The performance of LIP score in the screening of sepsis was evaluated by ROC analysis.**

**sFigure 2. The power of LIP tool to screen sepsis when the LIP score was greater than or equal to 2,3 and 4, respectively.**

**sTable 1. Comparison of AUROC between each combined biomarkers in the screening of sepsis.**

**sFigure 1. The performance of LIP score in the screening of sepsis was evaluated by ROC analysis.**


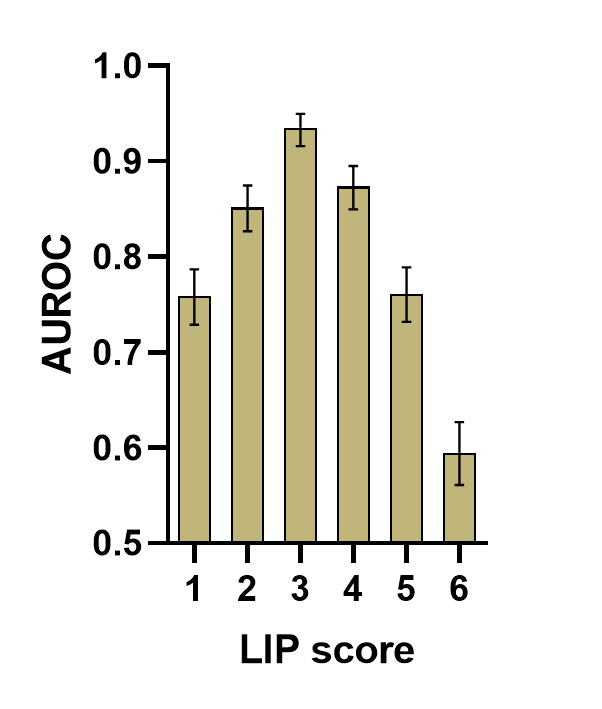


When the LIP score is greater than or equal to 3, the LIP score has the highest AUROC of 0.935 (95% CI, 0.916-0.950). Bar represents the 95% confidence interval of AUROC. AUROC: Area under the receiver operating characteristic curve. 95% CI: 95% confidence interval.

**sFigure 2. Sensitivity, specificity, PPV, and NPV corresponding to LIP scores of 2, 3, and 4.**

The screening power was higher when the LIP score >= 3 compared to the LIP score >= 2 or >= 4. Its sensitivity was 0.928(95% CI, 0.90-0.95), specificity was 0.941(95% CI, 0.915-0.961), PPV was 0.941(95% CI, 0.916-0.958), and NPV was 0.929(95% CI, 0.903-0.948). 95% CI: 95% confidence interval, PPV: Positive predictive value, NPV: Negative predictive value.

**sTable 1. Comparison of AUROC between each combined biomarkers in the screening of sepsis.**

|  | All patients | | Pulmonary infection group | | Non-pulmonary infection group | |
| --- | --- | --- | --- | --- | --- | --- |
|  | Difference  between AUROC | *P* value | Difference  between AUROC | *P* value | Difference  between AUROC | *P* value |
| LI *vs* LP | 0.018 | 0.011* | 0.008 | 0.625 | 0.021 | 0.009* |
| LI *vs* IP | 0.014 | 0.023* | 0.003 | 0.869 | 0.004 | 0.388 |
| LI *vs* Triplet | 0.011 | < 0.001* | 0.040 | 0.001* | 0.008 | 0.022* |
| LP *vs* IP | 0.004 | 0.680 | 0.005 | 0.809 | 0.017 | 0.046* |
| LP *vs* Triplet | 0.029 | < 0.001* | 0.032 | 0.001* | 0.028 | < 0.001* |
| IP *vs* Triplet | 0.025 | < 0.001* | 0.037 | 0.005* | 0.011 | 0.001* |

LI, Lym plus INR; LP, Lym plus PCT; IP, INR plus PCT; Triplet, Lym plus INR plus PCT; * indicates a significant value, *P* < 0.05; *vs* refers to a comparison, Lym: Lymphocyte count, PCT: Procalcitonin, INR: International normalized ratio, AUROC: Area under the receiver operating characteristic curve.
